# “It Helped Me Feel Like a Researcher”: Reflections on a Capacity-Building Program to Support Teens as Co-Researchers on a Participatory Project

**DOI:** 10.1177/07435584231176992

**Published:** 2023-06-01

**Authors:** Kendra Nelson Ferguson, Stephanie E. Coen, Jason Gilliland

**Affiliations:** 1 Western University, London, ON, Canada; 2 Children’s Health Research Institute, London, ON, Canada; 3 Lawson Health Research Institute, London, ON, Canada; 4 University of Nottingham, Nottingham, UK

**Keywords:** youth development, youth engagement, teen co-researchers, qualitative research, youth participatory research

## Abstract

The inclusion of youth voices in research relating to their own daily environments, wellbeing, and development is increasingly recognized as essential to ensuring rigor and success in mobilizing community change. Few studies have qualitatively examined youths’ experiences and perceptions in participatory roles. This paper presents insights and lessons learned from a capacity-building program designed and delivered as part of a youth participatory research project, Teens Talk Vaping. Teens Talk Vaping took a by-youth-for-youth research approach to co-produce research about teen vaping to inform evidence-based vaping education materials. The capacity-building program was developed to equip seven teens from the Human Environments Analysis Laboratory Youth Advisory Council (four teen girls, three teen boys; Mage = 17.3) with qualitative research skills to contribute as “teen co-researchers” to all phases of the project, from conceptualization through to dissemination. The teen co-researchers were interviewed at four key phases of the program: qualitative research principles and approaches, data collection, data analysis, and overall reflections. Using thematic analysis, findings revealed the positive implications and practical limitations of the capacity-building program, which may support other academics engaging in participatory methodologies with youth and contribute toward the improvement and enrichment of participatory research opportunities for youth.

## Introduction

The inclusion of youth voices in academic research relating to youth is increasingly recognized as essential to ensuring rigor and success in mobilizing community change (Checkoway & Richards-Schuster, 2003; Head, 2011; Kim, 2015; Kothari & Wathen, 2013). A participatory approach helps to warrant the trust of youth participants as it creates a sense of comfort when engaging in dialogue with their fellow peers, often generating more reliable data (Kirshner et al., 2002; Powers & Tiffany, 2006). Further, youth who are involved as part of the research team may benefit from enhanced social connectedness, enriched social development, feelings of community belonging and ownership, and the opportunity for intergenerational collaboration (Osborne et al., 2017; Powers & Tiffany, 2006). Youth can also exercise their social and political rights by engaging in participatory citizenship and democratic participation by practices of decision-making, independence, responsibility, and accountability (Matthews & Limb, 2003). This can provide youth, traditionally an under-represented and often marginalized group, with “new strategies of civic engagement which will awaken them to community conditions, enable them to reflect upon the root causes of problems, and motivate them to take action in a civil society” (Checkoway & Richards-Schuster, 2003, p. 23).

Academic researchers are aware of the benefits of youth inclusion (Arunkumar et al., 2019; Freeman & Aitken-Rose, 2005); however conceptual, methodological, and practical challenges exist in defining their role (Arnstein, 1969; Arunkumar et al., 2019; Kim, 2015). Commonly reported are the power imbalances and tokenistic practices that can result from youth-adult partnerships, often creating an illusion of participation (Arnstein, 1969; Lauria & Slotterback, 2021; O’Connor, 2013). Misconceptions and negative assumptions (e.g., excessive time commitment, issues around data quality) about youth involvement can limit how adults approach youth engagement in the research process (Nairn et al., 2007; Schäfer & Yarwood, 2008). Such constrained engagement contributes to power asymmetries between adults and youth, with “young people expressing feelings of inferiority and lack of decision-making power” (Arunkumar et al., 2019, p. 3; Osborne et al., 2017). Consequently, youth participation in research is vastly dependent on how inclusive adult researchers are (Kim, 2015).

Various frameworks have been adapted from Arnstein’s (1969) typology of a ladder of youth participation in research. Most notable is the rendering of a youth-devised model of participation by Arunkumar et al. (2019), which is attuned to youths’ goals and needs. Conceptualized as a “rope ladder” (Arunkumar et al., 2019), this model emphasizes the mutual benefits and exchanges of adult-youth partnerships, and is based on seven key premises: (1) ensuring genuine participation through youth agency, (2) being inclusive of diverse youth, (3) creating a flexible and adaptable approach to account for everyday life, (4) considering the contextual factors that influence dynamics of participation, (5) transparency through decision-making processes so that adults remain accountable to youth, (6) creating trustful partnerships, and (7) addressing the complexity of experiences, needs, and visions of youth-adult partnerships. Still, when youth are involved in research in participatory ways, there are often limits to the scope of their participation (Anyon et al., 2018; Jacquez et al., 2013). More recently, we have seen the advent of methodologies that seek to include youth *as researchers*. This takes various forms and has been labeled with various terms, including Youth Participatory Action Research (YPAR), youth-led research, youth engagement, youth leadership, youth advocacy or youth decision-making, youth involvement, youth empowerment, Community-Based Participatory Research (CBPR), and Youth-Adult Partnership (Branquinho et al., 2020).

Some researchers have suggested guiding principles for these forms of participatory research to fully integrate youth perspectives and experiences in research. For example, Rodriguez and Brown (2009) argue that YPAR programs should follow three principles to mitigate any potential power imbalances in the context of youth research. First, research topics should be grounded in youths’ lives and concerns. Second, the research must be participatory where youth are collaborators and engaged both methodologically and pedagogically. Third, research is transformative to ensure knowledge and practices benefit youths’ lives and their communities. Further, Ozer and Douglas (2015) acknowledge the key processes of YPAR, where a reciprocal adult-youth partnership with shared decision-making and participation is vital. This necessitates training in research design and methods, critical and strategic thinking about creating social change, and the opportunity to build alliances with stakeholders. As the inclusion of youth voices and decisions in the research process can allow for more effective community-level outcomes (i.e., appropriate practices, policies, research programs) that directly relate to the lives of this demographic, it is important to consider these principles, processes, and practices in YPAR.

The ethical imperative and benefits of youth involvement in research are evident; however, few studies have qualitatively examined the experiences and perceptions of youth who are engaged in participatory roles. Therefore, this paper was guided by the following research question: What insights and lessons can be shared from a capacity-building program that was designed and delivered to support a group of teens as “co-researchers” on a youth participatory research project? Our goals are to support other academics engaging in participatory methodologies with youth and to ultimately contribute toward the continual improvement and enrichment of participatory research opportunities for young people. We use the term “teen co-researcher” to refer to a participatory research role where youth are members of the research team and are provided with capacity-building support and ongoing mentorship to foster their research skills development and meaningful engagement across all phases of the project, from conceptualization through to dissemination. In what follows, we introduce the research context, our Teens Talk Vaping project. We then describe our capacity-building program, followed by the results of our interviews with our teen co-researchers about their experiences with the program. We conclude by drawing out ways that youth co-researcher roles can be improved by providing recommendations for researchers looking to adopt participatory approaches.

### The Teens Talk Vaping Project

Teens Talk Vaping was a qualitative integrated knowledge translation (iKT) study focused on understanding the everyday contexts of vaping in the lives of teens in Canada, funded by the Canadian Institutes of Health Research (CIHR). As a methodology, iKT centers equal partnership among researchers and individuals or groups who can use the knowledge generated through the research (also called knowledge users) to produce more relevant, applicable, and usable research evidence (Canadian Institutes of Health Research [CIHR], 2015). In applying this partnership principle to research on teen vaping, our primary knowledge user partners were teens, along with other stakeholders who could use our findings to support their teen vaping prevention and education efforts (including health units and school boards). Fully embracing an iKT approach requires knowledge user involvement in all phases of a study. For Teens Talk Vaping, this began with project conceptualization, whereby vaping was identified as a priority topic by the Human Environments Analysis Laboratory Youth Advisory Council (HEALYAC) at Western University (London, Ontario, Canada), where the study was based. Established in 2018, the HEALYAC—comprised of high school and early year university students from across the greater London, Ontario area—actively advises on all aspects of HEAL’s research on children and youth. When established, a Terms of Reference document outlining the values and structure was created, emphasizing diversity as one of the core values. A diverse group of youth who bring varied experiences, perspectives, and backgrounds to the table is central to advancing the mission of the HEALYAC. The HEALYAC shared critical input during the development of the project, including the grant development stage. The primary method of data collection involved online focus groups led by the teen co-researchers. This project sought to generate evidence on youth vaping, by-youth-for-youth, to inform youth-devised vaping education resources, where teens’ involvement was vital for the success of the research. We advanced a youth-devised model of youth participation based on the “rope ladder” model (Arunkumar et al., 2019) to develop a study focused on a health issue that our teen collaborators identified as important, and by developing a participatory research design that included teens as team members.

Teen co-researchers were recruited to the project from the HEALYAC, which comprised 18 members during the 2020/2021 term. Members of the HEALYAC were invited to apply for positions as paid co-researchers on the Teens Talk Vaping project. Ten members applied; however, after discussion about roles, responsibilities, and time allocation for the position, three applicants did not have the capacity to fulfill the role due to school and extracurricular commitments. This resulted in a team of seven co-researchers (four identified as teen girls, three identified as teen boys; average age 17.3 years; four high school students, three first-year university students), who came from a group (the HEALYAC) that was already constructed with diversity in mind. Teen co-researchers were paid according to the University pay scale for undergraduates, which was $17.38/hr (minimum wage in Ontario at the time was $15/hr), for 5 hr per week. Our weekly/biweekly online training sessions took an hour to an hour and a half to complete, with the remaining time allocated for teen co-researchers to complete other tasks related to the project. As part of our participatory principles, these roles were conceptualized as paid to value the teen co-researchers’ time and contributions as full team members (Merves et al., 2015). This mitigated against teens’ financial circumstances as a barrier to participation, and ensured equity across the team, as the rest of the investigative team of adult researchers’ time was accounted for within the context of their academic jobs. We developed a bespoke capacity-building program to equip the teen co-researchers with the qualitative research skills to contribute across all phases of the project, from data collection through to the dissemination of findings. More specifically, the training guided teens in taking leadership roles in data collection (e.g., online focus groups) and analysis (e.g., thematic analysis) and supported their development of knowledge translation products (e.g., short film, social media campaign) to share key research findings back to teens and other knowledge users.

### Capacity-Building Program for Teen Co-Researchers

Our capacity-building program took place between August 2020 and June 2021 and was made up of four parts. Two qualitative adult researchers (K.N.F & S.E.C) co-developed and co-led all training sessions, and a PhD student, with experience leading creative interdisciplinary design projects, facilitated the creative product concept development in the dissemination phase. Given the COVID-19 pandemic context we were operating in at the time, our entire program took place online on either Zoom or Microsoft Teams. Part 1, Qualitative Research Principles and Approaches, consisted of 11 training sessions focusing on introducing qualitative research methodologies, key ethical and practical considerations, gender considerations in data collection with specific attention to Canadian Institutes of Health Research’s Sex- and Gender-Based Analysis Plus Policy (CIHR, 2023), and the development of our focus group guide. The qualitative training material was supported by literature such as Braun and Clarke’s (2006, 2013) work, with readings integrated into sessions including Foster-Fishman et al.’s (2010) youth participation for social change paper and Hopkins’s (2007) work on thinking critically and creatively about using focus groups in human geography. In keeping with our iKT model, we worked in collaboration with the teen co-researchers to develop our focus group guide, factoring in how gendered identities, social relations/hierarchies, and intersectional structures (e.g., race/ethnicity, socioeconomic circumstances) are implicated in teen vaping practices. Once the focus group guide was finalized, we brainstormed potential obstacles within focus groups pertaining to intragroup dynamics (e.g., ingroup bias, groupthink) and ways to mediate such issues to ensure an inclusive and comfortable environment for dialogue. Several mock focus groups were then conducted as a group, and the teen co-researchers felt prepared to move on to Part 2: Data Collection. Using our co-developed focus group guide, each teen co-facilitated one online focus group (together with an adult researcher, either K.N.F or S.E.C.) with participants aged 13 to 19 years from across Canada. Following the completion of data collection, Part 3: Data Analysis Training began, and this training spanned across 11 sessions. After co-researchers completed focus group transcription and verification, an introduction to principles and procedures of thematic analysis (e.g., sorting, synthesizing, reducing) were discussed, guided by Auerbach and Silverstein’s (2003) work. The teen co-researchers were responsible for finding similarities across the data through open coding, thematic clustering, and dialectical discussion. Teen co-researchers began by coding their focus group individually (i.e., code names, defining codes, providing relevant text/quotes). Co-researchers were then paired to discuss codes, similarities, and differences across the data. Each pair created a combined coding table with common codes/themes between focus groups. Lastly, the group (teens and adults) worked together to reconcile commonalities and differences across all groups and created a coding master list. Working together as a team allowed co-researchers to reflect on the experiences of their peers, helping adult researchers gain a more grounded portrayal of the social context through their interpretation. After the data were analyzed, teen co-researchers moved on to Part 4: Transforming Data into Action (five sessions). Here, the co-researchers engaged in various creative workshops to prepare for the development of a creative product to disseminate key findings back to teens.

## Methods for Exploring the Capacity-Building Program

### Participants

Once our capacity-building program commenced, teen co-researchers were informed by the adult researchers that they had the opportunity, if interested, to participate in a study to evaluate our capacity-building program and the teen co-researcher experience. Teen co-researchers were made aware that their participation was voluntary, and that they could withdraw at any time, without any impact on their role in the project and/or with the Human Environment Analysis Laboratory. To protect individual identities, a qualitative researcher affiliated with the laboratory but not affiliated with the Teens Talk Vaping project was asked to facilitate recruitment, conduct interviews, and to transcribe and anonymize transcripts. This researcher reached out via email to each co-researcher with a letter of information detailing the purpose of the study and plans for interviews to be conducted across four timepoints throughout their time as co-researchers on the Teens Talk Vaping project. Those who were interested in completing interviews provided consent via Qualtrics. Participants were assigned a unique ID to anonymize their comments. All interview transcripts were cleaned of any potentially identifying information, including gender, before being shared with K.N.F. for analysis.

### Data Collection

Study approval was attained from Western University’s non-medical research ethics board (#116261) and the University of Nottingham’s School of Geography Research Ethics Committee. Once consent was obtained, a Doodle Poll was sent to each teen co-researcher to schedule their interview times, a process that was repeated at each interview time point. As mentioned, the interviewer was not affiliated with the Teens Talk Vaping project. Interviews were conducted online via Zoom. A semi-structured interview approach was used, adopting a conversational interview method to allow for some autonomy and flexibility, enabling unconstrained dialogue but also to encourage rich descriptions from the co-researchers (Patton, 2014), thus deepening communication with each participant.

Interview guides were developed to explore teen co-researchers’ experiences during each part of the capacity-building program, including qualitative research principles and approaches (Interview 1), data collection through focus groups (Interview 2), data analysis training and experience (Interview 3), and overall reflections on becoming a teen co-researcher (Interview 4; see Appendix A for interview guides). Collectively, 24 interviews were conducted, ranging between 14 and 30 minutes, averaging 21 minutes. Since co-researchers devoted 5 hours per week to the project on top of academic responsibilities and extracurricular commitments, we designed interviews within the constraints of their daily lives by keeping interviews to a maximum of 30 minutes, but allowing additional time if conversation were to continue beyond (none did). Each interview was audio recorded and transcribed verbatim by the interviewer and then verified by the teen co-researcher interviewee, resulting in a total of 171 pages of transcripts (11-point font, single-spaced) across four sets of interviews. Not all teen co-researchers participated in each round of interviews. See Table 1 for a breakdown of participation, interview length, and pages of transcripts per timepoint.

**Table 1.** Breakdown of Participation, Interview Length, and Pages of Transcripts per Timepoint.

	Interview 1: qualitative research principles and approaches	Interview 2: data collection	Interview 3: data analysis	Interview 4: overall reflections
Number of participants	6	5	7	6
Mean interview length in minutes	24.7	20	19.1	23.2
Pages of transcripts (11-point font, single spaced)	45	40	40	46

### Data Analysis

The aim of the analysis was to develop categories from the interview data to gain an understanding of program effectiveness in meeting the needs of the teen co-researchers to feel well-equipped and supported in their roles, as well as how the teens experienced their role as co-researchers. An inductive thematic approach was used to guide the analysis, as the analysis was reflective of the content of data and not pre-existing theory (Braun et al., 2015). The analysis followed Braun and Clarke’s (2006) six-phase framework. Following interview transcription and verification, the lead author read and re-read transcripts to fully comprehend the content of interactions, assigning preliminary codes to the data (phases 1 and 2). Codes were then compared and organized based on common themes (phase 3). Initial themes were reviewed and refined (phase 4), allowing for clear definitions within the data to develop, enhancing each theme (phase 5).

The study was guided by a relativist ontology (no one reality can exist), with a subjectivist epistemology as experiences rely on subjective interpretations of reality, where social constructions vary from individual to individual. Sparkes and Smith’s (2009) relativist approach to conceptualizing rigorous qualitative research was applied, meaning quality indicators are drawn from a list of characteristics that are not fixed. For this study, the list included five markers of quality identified by Tracy (2010) to guide our rigorous approach, as follows. First, the topic is worthy and provides relevant, significant, and interesting insights and lessons learned from a youth participatory research project. Second, credibility was accomplished through the use of a critical friend and member reflection (elaborated below). The interviewer—a skilled qualitative researcher familiar with the data collected—acted as a critical friend by engaging in critical dialogue with the lead author to explore alternative perspectives of the data, enhancing its trustworthiness. This process allowed for the lead author and interviewer/critical friend to challenge interpretations of the findings to deepen understanding, increase scope, and ensure consistency of results (Smith & McGannon, 2018). It should be noted that the interviewer was not only a qualitative researcher, but also an expert in children’s health research and community engagement, allowing opportunity to bring the interviewer’s critical reflections into our analysis process. From these critical friend dialogues, some themes were combined, removed, and refined to capture the nuances of the data more robustly. For example, “supportive environment,” “flexibility,” and “efficient training” were merged into one theme, “prepared and supported,” as critical deliberations identified sufficient overlap to fit under one theme. Once results were finalized, a summary of findings was sent to each co-researcher for member reflection. Member reflection aims to explore each participant’s interpretations of the findings to fill any potential gaps in the results (Smith & McGannon, 2018). One out of seven co-researchers provided extra dialogue and insight, which the interviewer/critical friend added to the analysis to safeguard participant anonymity. Third, Tracy (2010) indicates that results should provide an important contribution to researchers looking to adopt similar concepts and methodologies. Therefore, we aimed to showcase how youth engagement and involvement in the research process may contribute to positive youth development. Fourth, resonance was attained as findings have the potential to influence or impact readers and/or researchers to intuitively transfer such findings to their own situations. Finally, sincerity was achieved through self-reflexivity in relation to values, positionality, and project role. For example, the lead author, who co-led the capacity-building program and developed the interview guides, engaged in ongoing reflection on how her involvement and position in the project could shape research interpretations. This included critically considering her adult positionality, while still occupying a position proximate to students (i.e., teen co-researchers), having recently completed a Ph.D. and this being her first lead teaching role at the time. The lead author further drew on the use of a critical friend and member reflection to deepen her reflexive praxis.

## Results

To aid researchers in applying our findings, we present our thematic results within the frame of two overarching categories: positive implications and practical limitations. Positive implications include themes related to perceptions of being prepared and supported, the valuable skills attained, as well as reflections on the by-youth-for-youth research approach, team dynamics, and the process of becoming a teen co-researcher. Practical limitations include themes covering the barriers in relation to program delivery (e.g., the use of an online platform) and the time commitment of data collection and analysis. Each theme is accompanied by participant quotes to exemplify perceptions of the capacity-building program and teen co-researchers’ experiences. Participant codes have been used to maintain participants’ anonymity. Table 2 provides a breakdown of themes and the number of participants reporting each theme per interview.

**Table 2.** Interview, Category, Theme, and Number of Participants Reporting Each Theme.

Interview	Category	Theme	Number of teen co-researchers reporting each theme
Interview 1: qualitative research principles and approaches	Positive implications	Prepared and supported	6/6
		Building valuable skills	5/6
		Team dynamics	6/6
	Practical limitations	Online platform	5/6
		Time commitment	2/6
Interview 2: data collection	Positive implications	Prepared and supported	5/5
		Building valuable skills	5/5
		Participant comfort	4/5
	Practical limitations	Online platform	3/5
Interview 3: data analysis training and experience	Positive implications	Prepared and supported	7/7
		Building valuable skills	7/7
		Team dynamics	7/7
		Becoming a teen researcher	7/7
	Practical limitations	Time commitment	6/7
Interview 4: overall reflections	Positive implications	Prepared and supported	6/6
		Building valuable skills	6/6
		Becoming a teen researcher	6/6

### Positive Implications

#### Prepared and Supported

Feeling prepared and supported was a salient theme across each round of interviews. Collectively, in the first round of interviews, and I did a bunch of video calls before pretending to be people all teen co-researchers found the qualitative research principles and approaches training to effectively prepare them for data collection. One participant said, “all around, I learned a lot. I learned everything I needed to know. All questions I had were informatively answered, so you know, I just had all the information and knowledge I needed” (P05). P06 also revealed that “when we were practicing in a session or in a meeting, we would run a lot of situations. They tell us how we could do stuff better, if it was good and if it was not, it was very insightful.” It was also stated that “everyone felt confident, and I know I felt confident [by] the time we got to data collection” (P02). The co-researchers also found the training environment to be supportive. One participant said, “when exam week [was] coming, [the adult researchers] were very considerate by giving less and packing it more when we’re starting a semester” (P04). These comments accentuate the importance of creating a team culture of care and flexibility for team members to feel valued, heard, and respected.

Additional remarks were made during the second round of interviews, where it was apparent that all participating co-researchers (*n* = 5) felt they were efficiently prepared and supported during data collection. In relation to focus group training and mock focus groups, one co-researcher mentioned, “I felt very ready [and] prepared. We did a lot of practicing before, and the other co-researchers and I did a bunch of video calls before pretending to be people we were talking too, so I felt prepared” (P01). Another participant acknowledged that “[the adult researchers] were always supportive, they [were] always encouraging and complementing [and] that was a really efficient way to boost morale and things like that” (P05). Another participant added remarks regarding program delivery and made the point that although “we learned everything digitally, we were about as prepared as I think we would have been [and] if we weren’t in COVID, the same result would have occurred in person” (P06). There was a shared sentiment that a supportive and encouraging training environment led to co-researchers effectively executing their focus group facilitation roles.

Further emphasis was placed on feelings of support and preparedness when co-researchers were asked about data analysis training in round 3 of the interviews. One co-researcher said, “it was kind of interactive. They would have an example of a coding sheet or template on the screen, but then we do it with them, which was very helpful in getting us started to do our coding” (P03). They added, “I feel like they were really open with us asking questions no matter how farfetched they were, they were just very accepting” (P03). Another participant felt that the progression of the training helped them to comprehend instructions: “having those little step by step increases [the] kind of learning and then also the live example, the slide show examples of how to code, that helped a lot” (P05). By incorporating interactive training sessions with gradual progressions, the co-researchers noted that this method of guidance gave them confidence in their abilities moving forward.

During the fourth round of interviews, the co-researchers (*n* = 6) recounted a sense of comfort and security as they reflected on the entire capacity-building program. One participant said, “everyone was perfectly on the same page. It was a very secure process. I think they did everything great, and I compliment everyone who worked with us that was helping us to get through it all” (P06). Another participant discussed how the adult researchers’ encouragement was helpful: “they took us from knowing nothing, or almost knowing nothing to a point where we were very comfortable and we just wanted to keep doing it, so it was very, they were very encouraging. They helped a lot” (P07). Lastly, one participant mentioned, “you know the materials presented in an organized fashion like the training, it was clear. What we had to learn and then you know every situation, they really prepared us well for all those possible circumstances” (P05). Taken together, these comments shine light on the importance of developing a secure, flexible, and supportive team environment so teens feel comfortable in their skill development and utilization.

#### Building Valuable Skills

Through each round of interviews, teen co-researchers spoke to the valuable skills attained through each part of the capacity-building program. During the first round of interviews, most teen co-researchers (*n* = 5) spoke about how the qualitative principles and approaches training helped them to learn more effective communication skills. One co-researcher detailed, “to be a better leader is to use my voice, so this was a better like branch into enhancing [my communication skills] and working on that more intimately” (P03). This teen went on to say, “it was nice to be able to know how to communicate in a way that was like comforting but also helpful for research” (P03). Relatedly, another co-researcher said, “I think that was just a general skill you know, people might be shy, reserved, especially online so just being able to kind of get that communication going” (P05). Added nuance was made to this theme during the second round of interviews as co-researchers (*n* = 5) revealed that participating in data collection impacted their communication and leadership skills. This is accentuated in the following quote:

Being able to navigate a conversation in the way of like a leadership role. [The adult researcher] and I, we had to, you know, pivot, sometimes or really had to listen to what the [participants] were saying so we could ask the appropriate questions that connected to what they were saying. I thought that was interesting and a great thing that I learned. (P01)

Another participant added, “[I think it is important] to get used to being put in unfamiliar situations and things like that, [while knowing] how to properly respond and then do your best despite any difficulties that you may have” (P05). That same participant went on to say, “being online and everything so kind of building on those communication skills in, in a new environment I think is really beneficial” (P05). The teen co-researchers were able to identify benefits to their learning and professional development that were valuable beyond the scope of their collaboration on the project.

Building on this, all co-researchers expressed that they developed more patience, teamwork, empathy, and critical thinking skills during the third round of interviews. For example, one co-researcher mentioned, “I like to be very efficient because time is precious, but this showed me that there’s cases in which it won’t be quick and you need to be aware and be patient so this definitely helped me with that” (P06). Another said, “being able to collaborate with others [and] understanding each other’s thoughts was really important, so being able to improve teamwork [and] collaboration skills” (P05). Furthermore, one participant detailed how engaging in qualitative research allowed them to get in tune with their emotions. This sentiment is captured in the following quote:

You want people who are working in health to be empathetic and to be able to really resonate with the general population, so I think exposing people to qualitative research will do what it did to me, and it helps you get more in tuned with your own emotions, which is beneficial for future health care workers. (P02)

During the fourth round of interviews, participants (*n* = 6) reflected on how the valuable skills gained can, in fact, transfer to other areas of their lives. These skills included working well with diverse teams, valuing different points of view, finding confidence when speaking in front of others, being able to see the bigger picture, developing deeper critical thinking skills, being able to share their thoughts and opinions, and maintaining organization. Some of these skills are articulated in the following quote: “This allowed me to work with people of different ages, ideas, abilities, and that is definitely important [and] helped me prepare for working in a larger group where ideas might be different and how to compromise and work together” (P06). Another co-researcher pointed out:

Being able to look at things from a different point of view, like I guess putting yourself in the place of your peers that may not have the same opinions as you or knowledge as you regarding something like vaping. I think that was a new skill, I guess slightly challenging but something that we had to do. (P01)

In addition, one participant spoke about how the training helped with public speaking:

I’m not the biggest speaker and I remember when I showed my family [a video of me public speaking], they were shocked and saying, “you’re speaking so clearly, you’re talking like a professor” and they were teasing me a bunch. But I think that’s very noteworthy of how this has helped me become a better speaker. (P03)

These statements convey how meaningful the capacity-building program was to co-researchers. As described, the valuable skills attained enhanced their agency/leadership as well as interpersonal skills and knowledge, underscoring the positive impact of youth participatory research.

#### Participant Comfort

The next theme, participant comfort, was only specific to the second round of interviews when participants recounted their experiences with data collection. It should be noted that our focus group participants were invited to preselect a non-identifying pseudonym, and that video was disabled for everyone except the facilitators to protect intra-group anonymity and confidentiality. Most of the co-researchers (*n* = 4) concurred about how the by-youth-for-youth approach, along with the anonymous nature of online focus groups, may have enabled participants in the focus groups to feel more comfortable in disclosing personal experiences and perceptions on the topic. One co-researcher said, “I was a bit surprised by the comfort in which the participants had. They were very quick to talk, feel comfortable, and display information. Obviously, they were anonymous, but there was a sense of comfort within the group” (P06). To add, P05 said, “they put a lot of detail behind their answers, we were getting a lot of discussion and material, we didn’t need to probe much into questions. I wasn’t personally expecting [such] detailed answers, so that was very nice.” Another co-researcher shared that “[the] philosophy [is] so unique from other research in that we have the by-youth-for-youth kind of philosophy. I think it definitely does make a big impact and it makes the participants feel more comfortable” (P01). This theme is a nod to our research approach and gives readers a glimpse at how the teen co-researchers felt our methodology contributed to the success of the project and the richness of the data collected.

#### Team Dynamics

For the first and third round of interviews, teen co-researchers had a chance to discuss the team dynamics of the cohort of teen co-researchers and how they experienced aspects such as group size and working together. All teens who participated in the first round of interviews (*n* = 6) felt as though the group size was effective: “it was a great size. I think it worked well and the fact that our focus groups are done and we’re on track with everything I think is sort of proof. More people might have made it complicated” (P06). Further, P02 added, “if it was smaller the group dynamic would have been awkward, and if it was larger than it would have been hard to get practice done and schedule things. So, I think it was a good size, perfect number.” During the third round of interviews, all co-researchers (*n* = 7) described their experiences with data analysis training and the benefits of working together as a team during the analysis process. One co-researcher said, “we were working together rather than learning from the [adult researchers]. I’m still learning from the [adult researchers], but there is more of an instance where we’re actually working together and not always just me learning from them alone” (P03). Similarly, P07 added, “we have high school students and like first year [university] students working together on this project, and I think we’re working really well. We are collaborating really well; we get our ideas across in a meaningful way” (P07). This theme highlights how program considerations, such as group size and intragroup relationships, may be beneficial for future implementation.

#### Becoming a Teen Co-Researcher

The theme “becoming a teen co-researcher” was echoed across all co-researchers during rounds 3 and 4 of the interviews. It was evident that their involvement in the project was enjoyable and rewarding. It was during data analysis that the co-researchers began to feel like skilled researchers, which was emphasized during round 3 of the interviews. At this point, they had completed their data collection training, facilitated online focus groups, and were immersed in qualitative data analysis, where they were able to reflect on all the work they had done to that point. This is highlighted in the following quote:

It helped me feel like a researcher, like at the beginning we were kind of training to become researchers and then at the focus group that’s kind of when I started to feel like really part of this project. And like I’m researching and then analyzing the data really solidified that feeling because I’m actually looking at the information and connecting the dots and organizing my thoughts and stuff like that. (P07)

Another teen co-researcher said, “this process definitely made me feel like a researcher and [the adult researchers] constantly reminded us of how much we’ve grown as researchers and it would shock me like, they’re actually making us researchers, that’s crazy” (P03). Adding to this, P01 added, “I definitely felt like a real researcher, which is nice. I will be continuing researching in my next, you know, 10 to 30 years, you know, part of my career so it’s nice [when you are] the age of like 17/18 [and] you can feel like you’re actually researching.” At this point in time, their personal growth and involvement became more evident, recognizing their impact on the project as they continued to engage in each phase of the project.

To conclude, the teen co-researchers conversed about how rewarding the process of becoming a teen co-researcher was during the fourth round of interviews. One co-researcher said, “when we started getting involved in the process, I felt so proud of the work that was done [because] we were actually invited to work on all of the project” (P04). Similarly, another co-researcher documented how “experiences like this for people [our] age don’t come often and aren’t appreciated often when they do” (P06). That same participant elaborated by saying, “moments like this will give life experience that might open the door in the future for some unknown reason, and being able to do something productive is important, especially if you have the opportunity to do it” (P06). To add, P05 mentioned that “it was great being part of the team from start to finish. Seeing the development of the ideas just that initial idea to what it’s become now. It’s been a journey and great seeing everything kind of click together.” These comments encompass the joy and fulfillment each teen experienced as they evolved into a researcher.

### Practical Limitations

#### Online Platform

Due to the COVID-19 pandemic, our capacity-building program was conducted online, and some teen co-researchers felt it was a barrier to program delivery. During the first round of interviews, most of the teen co-researchers (*n* = 5) indicated that they would have preferred in-person training. One participant said, “it felt kind of lonely with the online environment [but] that wasn’t something that they could really control, but it was I think something that was a step back” (P03). Another participant added, “I feel like it would be really cool and so much more fun to actually train in person” (P04). The issues with being limited to the online environment persisted into our data collection phase as focus groups were facilitated online. In addition to identifying a need to connect with each other in-person, some teen co-researchers reported that this also mattered for their capacity to connect with research participants during the second round of interviews. One co-researcher pointed out:

There’s a missing element of being able to connect with the [participants] in person. You can’t expect [participants] to instantly jump on to a chat and be like, OK, I’m just going to fully, you know, be vulnerable, tell everything about myself in terms of even just like vaping as it is such a sensitive topic in itself as a as a teen, right? So, you couldn’t have expected them to be fully on and transparent with each other when they don’t even know each other. (P03)

Another co-researcher spoke to the communication challenges posed using an online platform, where participants may “speak over each other ‘cause they’re not sure when another person is going to speak” (P01). This same co-researcher further elaborated on how “that doesn’t [happen] in person [because] you can read the person’s body language and make eye contact” (P01). Although it was out of our control, feedback from the first and second round of interviews shows that in-person connection both with the research team and the focus group participants would have been preferred.

#### Time Commitment

During the first round of interviews, a couple of co-researchers felt the training took longer than expected: “it was a bit long. The ethics and the qualitative training. I had a lot of projects and tests around that time so there was a lot of juggling to stay on the ball, it was a bit arduous” (P06). Additionally, one co-researcher said, “it did take a little longer” but it was the “first time doing this so [the adult researchers] had nothing to go off of, but for everyone’s first time, I think we did a good job” (P01). These comments highlight the importance of striking a balance between the demands of their time commitment as co-researchers with the context of their lives as high school and early university students.

Further comments were made when co-researchers reflected on the data analysis process as part of the third round of interviews. When focusing on the data analysis phase of the program, most of the teen co-researchers (*n* = 6) detailed the significant effort that went into transcription, underestimating the time it would take to complete. One participant revealed, “I thought it was definitely really time consuming. I’m doing something, I’m doing it all in one sitting type of person. I said, OK, let’s do this [transcribing then] saw that it was like 35 pages” (P06). Another participant added, “how long transcript verification takes [and] how mentally grilling it is, it took a very long time” (P02), and P03 said “[transcribing] ended up being a lot more work than anticipated. It was an hour I think of audio and that takes a lot of time to just understand and interpret how people are saying things.” Such comments suggest that despite communicating about the intensity of the task and the time commitment involved, we could have done more to concretize these expectations for the teen co-researchers.

## Discussion

The purpose of this paper was to assess our teen co-researcher capacity-building program by exploring the perspectives of our teen co-researchers who participated in it. With growing evidence supporting teens’ involvement as requisite for research seeking to positively impact teens’ lives (Arunkumar et al., 2019; Checkoway & Richards-Schuster, 2003; London et al., 2003; Powers & Tiffany, 2006), it was critical to engage the knowledge and expertise of individuals who share the lived experiences of the research issue (Powers & Tiffany, 2006; Sabo, 2003). Thus, this project created research infrastructure where teen co-researchers’ concerns were prioritized. Such infrastructure considered the principles, processes, and practices central to YPAR while informed by a youth-devised model of participation (i.e., “rope ladder”; Arunkumar et al., 2019). Specifically, teens were provided with an outlet to think critically about their everyday experiences and connect these to a research project they had a stake in, motivating them to find solutions to the issues that affect them personally (Cammarota & Fine, 2008; Foster-Fishman et al., 2010; Ozer & Douglas, 2015; Rodriguez & Brown, 2009).

As adult researchers, the fruition of research tends to stem from passion and/or gaps in the literature, often relying on literature to steer research direction (Buck & Magee, 2017; Cahill, 2007). Taking a participatory research approach with members of our youth advisory council provided opportunity to incorporate their voices, strengths, and perspectives about issues relevant to their lives, which is central to YPAR (Arunkumar et al., 2019; Buck & Magee, 2017; Ozer & Douglas, 2015; Rodriguez & Brown, 2009). The epistemological framework of participatory research “challenges the normative production of knowledge by including excluded perspectives and engaging those most affected by the research process” (Cahill, 2007, p. 326). Using this approach encouraged teens to think critically about the social circumstances related to the research questions, allowing data to go beyond fact-gathering and report-writing by using the knowledge gained to transform data into something meaningful for the promotion of collective change (Kroeker, 1996; Powers & Tiffany, 2006; Reason, 2001). Therefore, scholars are pushed in new directions and challenged to go beyond prescribed ideas and methods, realizing the importance of alternative points of view (Buck & Magee, 2017).

In terms of the capacity-building program, it was apparent through all four rounds of interviews that teen co-researchers felt supported by their fellow peers and the adult researchers. This support played a vital role in the training and preparation for each stage of the project, especially when teens considered the flexible nature of the program, where transparency was valued, and timelines could be adjusted based on individual schedules and academic workloads (Arunkumar et al., 2019). As argued by Ozer and Douglas (2015) as well as Arunkumar et al. (2019), a critical process in situating youth as researchers is ensuring a reciprocal adult-youth partnership through research development. Although teen co-researchers were not asked directly about power dynamics, the notion of ideas being heard, valued, and implemented while “working together rather than learning from the [adult researchers]” (P03) speaks to the supportive nature of the training and our ethos of partnership. Sentiments reflect the constructive, encouraging, and trustful alliances built across the team, but also the methodological (i.e., engaged in research process [expertise, decision-making, authority]) and pedagogical (i.e., development of knowledge, transferable skills, critical thought) implications for such involvement (Arunkumar et al., 2019; Ozer & Douglas, 2015; Rodriguez & Brown, 2009).

Further, some teens discussed how the by-youth-for-youth approach contributed to the success of the project as research participants in our focus groups (co-led by teens) felt comfortable divulging personal experiences and insights. As noted in previous research, it may be easier for young people to gain the trust of other youth than adults, contributing to more valid and reliable results (Kirshner et al., 2002). Moreover, the by-youth-for-youth approach provided teen co-researchers with an opportunity that is not commonly presented to this demographic. As they reflected on the rewarding process, they also identified the transferable skills attained and the value of their participation from start to finish. This allowed them to see the project come together while feeling like a credible researcher. The outcomes and impact of teens’ involvement suggest some meaningful contributions to their growth and development. Consistent with our findings, emerging research points to the benefits of youth participatory research including increased interpersonal skills and knowledge (e.g., communication skills, teamwork, academic skills, job readiness, empathy), agency/leadership (e.g., confidence, voice, empowerment), high level cognitive processing (e.g., decision-making), social belonging, connectedness, and capital (e.g., relationships that may lead to future educational or employment opportunities), academic/career growth (e.g., public speaking, time management, organization, planning), emotional development (e.g., ability to identify and express emotions), and identity development (Anyon et al., 2018; Arunkumar et al., 2019; Kirshner et al., 2011; London et al., 2003; Rubin & Jones, 2007). These positive developmental outcomes for young people are promising precursors to future academic success and employment (Jenson & Bender, 2014). By allowing youth to exercise their participatory citizenship and democratic participation, we in turn motivate them to take action in civil society recognizing the importance of their voice in mobilizing community change (Checkoway & Richards-Schuster, 2003; Matthews & Limb, 2003). It is evident that the teen co-researchers found value in being part of the project and that the skills attained can reach beyond the project and to their everyday life. As such, by providing this structured opportunity while following the practices and processes central to YPAR, we begin to understand the potential impact these approaches have on youth development. Based on the positive implications and practical limitations of these findings, recommendations for researchers looking to adopt participatory approaches are offered in Box 1.

**Box 1.** Recommendations for Researchers Looking to Adopt Youth Participatory Approaches.

• Engagement: Our findings highlight the importance of youth having their voices heard, valued, respected, and supported. Engaging teen co-researchers at each stage of the project ensured connection and inclusion, which was critical for the project’s success.
• Supportive environment: To foster a supportive environment, we placed emphasis on building a positive youth-adult partnership that promoted trust, collaboration, and openness. Based on our results, this support contributed to meaningful engagement as the teen co-researchers felt encouraged to share their thoughts and perspectives throughout the project.
• Training support: By providing in-depth training support, teen co-researchers began to feel confident in their roles. This was achieved by not rushing components of our capacity-building program and making sure to provide time for questions throughout each training session.
• Scheduling and open communication: Regarding the time commitment of participatory roles, it is critical to clearly communicate what their roles will entail, while being supportive and flexible in valuing individual schedules and academic workloads, realizing that schedules change for students each term with a need to be adaptable.

### Challenges and Limitations

There are some challenges and limitations that warrant discussion. The challenges drawn align with the practical limitations outlined by teen co-researchers. All phases of the project took place between August 2020 and June 2021, during which the COVID-19 pandemic presented lockdowns and/or quarantine orders in Ontario, Canada. Thus, these circumstances prohibited any in person contributions, as qualitative training, data collection, and analysis had to be conducted online. To add, data analysis was complex, and with the virtual environment, navigating and working together as a team contributed to some time constraints. Although teen co-researchers felt we navigated the situation as best we could, balancing societal disruptions while learning remotely as both a teen co-researcher and a student posed some difficulties.

In addition to the challenges outlined, there are limits to recognize in the scope of our analysis. First, not all teen co-researchers could partake in each round of interviews, due to scheduling or commitments outside of the project. Second, even though interviews were conducted by a qualitative researcher not affiliated with the Teens Talk Vaping project, it is still possible that some co-researchers could have felt the need to provide socially acceptable responses. We contend, however, that it is more likely that co-researchers’ largely favorable feedback reflects the deep level of positive meaningful engagement they experienced throughout the program. Next, when we consider generalizability, it is important to note that some of our teen co-researchers entered university during their involvement in the study, and consequently, these findings may not be transferable to working with youth who are not on a university-track or who are experiencing circumstances that may make sustained engagement difficult. It may also be useful to add that a different group of youth (e.g., those not on a university-track) may also have a different perspective on the acceptability/relevance of their engagement in research (e.g., if research is not perceived to be a skill they can feasibly employ in the future). Related to this, our analysis did not specifically consider how co-researcher experiences may differ across various sociodemographic (i.e., age, gender, race/ethnicity) factors because such data needed to be protected for anonymity purposes.

## Conclusion

Overall, little research has qualitatively documented the experiences, positive implications, and practical limitations of teens’ engagement in the research process from formulating research ideas to selecting methods, collecting data, analyzing data, and reporting and disseminating findings. Our capacity-building program allowed for our teen co-researchers to demonstrate knowledge, skills, capacities, and concerns for social change in youth populations. Findings highlight the importance of a by-youth-for-youth participatory approach and how teens’ expertise, voice, and lived experiences can strengthen perspective on the research issue. Through their participation, teen co-researchers were eager to be engaged in each phase of the project and their ability to think critically, utilize the skills acquired, and share their input led to the success of the project. These reflections help to pave a path for youth involvement in research and our findings contribute to future implementation of co-researcher roles. Future research should continue to explore and evaluate meaningful ways to engage teens in all phases of research.

## Appendix A

### Interview Guide 1: Qualitative Data Collection Training

#### Welcome

Thank you for taking the time to participate in an interview discussing your experience as a teen co-researcher for the Teens Talk Vaping project. My name is {insert name} and I am {insert affiliation}.

Today I’d like to hear about your experience as a teen co-researcher for the Teens Talk Vaping project. More specifically I’d like to hear about your experience with the qualitative training process. This would include thoughts on the training materials, the setup of the training, and your overall experience.

I want to emphasize that we really welcome you to be honest about your experiences, including things that didn’t work well for you or that you think could have been done better. What you share in this interview will have no impact on your role in the project or the HEALab and is really to help us evaluate our co-researcher methodology.

#### Guidelines

- Our conversation today will be audio-recorded, and I will be taking notes throughout the discussion.
- Your identity will remain confidential
- As we are asking about your personal experience and thoughts, there are no right or wrong answers. If you are not comfortable with answering a question, please let me know and we can move onto the next question.

At the end of the interview, we will review what we talked about and there will be time to add any ideas you may have.

1. Can you describe how the training materials were delivered to you?
  - How was this an efficient or non-efficient way to deliver the training?
  - How did you find the online training?
    i. Was it difficult?
    ii. Was it easy to follow?
  - What was the most useful training you received?
    i. Any specific topics come to mind?
  - Thinking about your weekly time requirements, including your meetings and take-home tasks, did you find the load to be too much, not enough, or sufficient?
    i. Did you feel you were able to manage your time efficiently or not?
    ii. Were you able to complete tasks on time with the according due dates?
2. What was the biggest learning that you took from this training?
  - Why is that learning important for training?
  - What was useful about it?
  - How did that help you?
3. How ready did you feel prior to start of data collection?
  - What specifically made you feel prepared?
  - Was there anything missing to ensure you felt prepared?
  - Did you feel like you had enough practice before collecting data?
  - Did you feel like the adult researchers provided you with enough time to feel prepared?
    i. What about feedback?
4. Can you describe the type of support you were provided throughout the training?
  - How was the communication between the adult researchers/trainers and yourself as teen co-researchers?
  - What kind of support and guidance did the adult researchers/trainers provide you with?
    i. Did you feel comfortable communicating with them?
    ii. Did you feel that you had sufficient encouragement/support from the adult researchers/trainers?
  - How was the communication between teen co-researchers?
    i. Did you find you worked well as a team?
    ii. What are your thoughts on the group size?
5. If you could change anything about the training what would it be and why?
  - How can it be improved upon for next time?
  - Is there anything missing from the training that should be included?
  - Is meeting once a week enough? Should it be shorter/longer?
  - Were there factors external to the training that supported or got in the way of your experience?
    i. Anything else going on in your life, outside of this job (e.g., school, work, etc.) that got in the way of you participating in the way you might have liked to?
6. Was there a point in the training, or after, when you started to see yourself as a researcher? Can you tell me about that?
7. Is there anything else you would like to add about your experience of the training program before we end the interview?

### Interview Guide 2: Data Collection

#### Welcome

Thank you for taking the time to participate in an interview discussing your experience as a teen co-researcher for the Teens Talk Vaping project. My name is {insert name} and I am {insert affiliation}.

Today I’d like to hear about your experience as a teen co-researcher for the Teens Talk Vaping project. More specifically I’d like to hear about your experience with data collection through online focus groups. A reminder, we really welcome you to be honest about your experiences, including things that didn’t work well for you or that you think could have been done better. What you share in this interview will have no impact on your role in the project or the HEALab and is really to help us evaluate our co-researcher methodology.

#### Guidelines

- Our conversation today will be audio-recorded, and I will be taking notes throughout the discussion.
- Your identity will remain confidential
- As we are asking about your personal experience and thoughts, there are no right or wrong answers. If you are not comfortable with answering a question, please let me know and we can move onto the next question.

At the end of the interview, we will review what we talked about and there will be time to add any ideas you may have.

1. Can you tell me about your experience with data collection?
  - How many focus groups were you a part of?
  - What did you enjoy about data collection?
  - What surprised you about data collection?
  - Was there anything you found difficult or that you didn’t enjoy?
2. How ready did you feel going into a focus group interview?
  - Did you feel like the training provided you with enough knowledge and experience to co-facilitate?
    i. If yes, please elaborate.
    ii. If no, please elaborate.
    What were your thoughts on conducting focus groups online?
    i. Do you think this was an efficient method considering the circumstances?
    ii. Could anything have been done to improve this method of data collection?
3. Can you describe the type of support you were provided during data collection?
  - How was the communication between the adult researchers/trainers and yourself as teen co-researchers?
  - What kind of support and guidance did the adult researchers/trainers provide you with?
    i. Did you feel comfortable communicating with them?
    ii. Did you feel that you had sufficient encouragement/support from the adult researchers/trainers?
4. What did you learn about yourself and your skills/capacities during data collection?
  - Did the experience prompt you to reflect on anything about your strengths? Your career development? Your role on the project?
  - What did you learn about young people as researchers and young people in general during data collection?
5. If you could change anything about the data collection process, what would it be and why?
6. What was the biggest learning that you took from this experience?
7. Is there anything else you would like to add about your experience with data collection before we end the interview?

### Interview Guide 3: Data Analysis Training and Experience

#### Welcome

Thank you for taking the time to participate in an interview discussing your experience as a teen co-researcher for the Teens Talk Vaping project. My name is {insert name} and I am {insert affiliation}.

Today I’d like to hear about your experience as a teen co-researcher for the Teens Talk Vaping project. More specifically I’d like to hear about your data analysis training and experience. A reminder, we really welcome you to be honest about your experiences, including things that didn’t work well for you or that you think could have been done better. What you share in this interview will have no impact on your role in the project or the HEALab and is really to help us evaluate our co-researcher methodology.

#### Guidelines

- Our conversation today will be audio-recorded, and I will be taking notes throughout the discussion.
- Your identity will remain confidential
- As we are asking about your personal experience and thoughts, there are no right or wrong answers. If you are not comfortable with answering a question, please let me know and we can move onto the next question.

At the end of the interview, we will review what we talked about and there will be time to add any ideas you may have.

1. Can you tell me about your experience with data analysis?
  - What did you enjoy about data analysis?
  - What surprised you about data analysis?
  - What did you find difficult and/or not enjoy?
2. Can you describe how the data analysis training was delivered to you?
  - What worked well/didn’t work well for you?
  - How was this an efficient or non-efficient way to deliver the training?
  - How did you find the online training?
    i. Was it difficult?
    ii. Was it easy to follow and did it make sense?
    iii. Did the training resonate with you?
3. What was the biggest learning that you took from the data analysis training?
  - Why is it important for youth who take this training to learn this?
  - What was useful about it?
  - How did that help you?
4. What did you learn about yourself and your skills/capacities during data analysis?
  - Did the experience prompt you to reflect on anything about your strengths? Your career development? Your role on the project?
  - What did you learn about young people as researchers and young people in general during data collection?
5. Is there anything else you would like to add about your experience with data collection before we end the interview?

### Interview Guide 4: Overall Reflections

#### Welcome

Thank you for taking the time to participate in an interview discussing your experience as a teen co-researcher for the Teens Talk Vaping project. My name is {insert name} and I am {insert affiliation}.

Today I’d like to hear about your experience as a teen co-researcher for the Teens Talk Vaping project. More specifically I’d like to hear about your overall reflections from start to finish.

#### Guidelines

- Our conversation today will be audio-recorded, and I will be taking notes throughout the discussion.
- Your identity will remain confidential
- As we are asking about your personal experience and thoughts, there are no right or wrong answers. If you are not comfortable with answering a question, please let me know and we can move onto the next question.

At the end of the interview, we will review what we talked about and there will be time to add any ideas you may have.

- To start, we would like to gain an overall reflection of your experience throughout the whole co-researcher experience. If you think back to the beginning, are you surprised about where you are now in terms of anything you’ve learned or skills you’ve gained?
  - What specific skills did you gain from the training?
    • How do you think these skills can transfer to other areas of your life?
  - The pandemic moved us online for our training—how did you find the online experience, and would you recommend it or not for future training? Why?
  - Is there anything you would have done differently?
  - Is there anything the adult researcher team could have done differently?
  - Do you feel that the adult researchers adequately prepared you for this experience? If so, how? If not, what could they have done to better prepare you?
  - How do you think your experience as a co-researcher has influenced your personal development, and going forward in your studies and/or career?
- If you had the opportunity to engage in a research project like this again, would you? Why or why not?
- What are the biggest things you learned about research from this experience?
- What are the biggest things you learned about yourself from this experience?
- What would you tell other teens about this experience?
- Is there anything else you would like to add about your overall experience before we end the interview?
